# Predictive factors and comparative analysis of surgical vs. endovascular treatment for spinal dural arteriovenous fistulas: a 22-year experience at a neurovascular and spine center

**DOI:** 10.1016/j.bas.2025.104335

**Published:** 2025-07-22

**Authors:** Mido Max Hijazi, Andreas Filis, Penelope Felgenhauer, Kay Engellandt, Sergio M.F. Romualdo, Dino Podlesek, Tareq A. Juratli, Ilker Y. Eyüpoglu

**Affiliations:** a Department of Neurosurgery, Technische Universität Dresden, Faculty of Medicine, and University Hospital Carl Gustav Carus, Fetscherstrasse 74, 01307, Dresden, Germany; b Institute of Diagnostic and Interventional Neuroradiology, Technische Universität Dresden, Faculty of Medicine, and University Hospital Carl Gustav Carus, Fetscherstrasse 74, 01307, Dresden, Germany

**Keywords:** SDAVF, Spinal arteriovenous fistula, Myelopathy, Endovascular treatment, Surgical treatment, Spinal cord edema, Spinal angiography

## Abstract

**Background:**

Spinal dural arteriovenous fistula (SDAVF) is a rare disease with insidious clinical manifestations that can often be overlooked on initial examination. Our aim was to identify predictive factors associated with favorable long-term outcomes and to compare the clinical results between surgical and endovascular treatment.

**Methods:**

A retrospective chart review was conducted of 81 patients with SDAVF who underwent either surgical (n = 70, 86.4 %) or endovascular (n = 11, 13.6 %) treatment at our hospital between 2002 and 2023, with a mean follow-up duration of 22.4 months.

**Results:**

A significantly greater proportion of surgical (S) patients (45/70, 64.3 %) showed improvement in the modified Aminoff and Logue Scale (mALS) between admission and last follow-up compared to endovascular (E) patients (3/11, 27.3 %; p = 0.043). There were no significant differences between the two groups over the clinical course (preoperative, postoperative, first, second, and third follow-up) in terms of mALS, American Spinal Injury Association Motor Score (ASIA-MS), back pain, or sensory disturbances. Incomplete or failed fistula closure occurred significantly more frequently in the endovascular group than in the surgical group (S: 3/70, 4.3 % vs. E: 5/11, 45.5 %, p < 0.001). The time interval between MRI-based diagnosis and treatment was identified as an independent predictor of long-term mALS improvement in the multivariate logistic regression analysis (0.978 (0.959–0.997), p = 0.026).

**Conclusions:**

Surgical treatment of SDAVF is generally safe and effective and shows superior outcomes compared to endovascular treatment; however, endovascular therapy remains an important option in special cases. Moreover, the time interval between MRI-based diagnosis and surgical or endovascular treatment plays a critical role in determining long-term clinical outcomes of SDAVF patients.

## Highlights

• Surgical treatment led to significant mALS improvement in SDAVF patients.
• Endovascular group showed a higher rate of treatment failure or incomplete closure.
• Clinical scores (ASIA-MS, pain, sensory) showed no group differences over time.
• Shorter time from MRI to treatment improved long-term neurological outcome.
• Surgery is safe and effective; endovascular therapy remains essential in select cases.

## 1 Background

Spinal dural arteriovenous fistulas (SDAVFs) are rare but the most common form of spinal vascular malformations (70–80 %)(Cesak et al., 2018; Krings et al., 2004; Black, 2006). SDAVFs usually emerge from a radiculo-meningeal artery with direct drainage into a radicular vein close to the spinal nerve root in the thoracolumbar region (Devalckeneer et al., 2023; Spetzler et al., 2002). Venous hypertension, which leads to chronic hypoxia and favors the development of congestive myelopathy, is thought to be the cause of the clinical symptoms (Hurst et al., 1995; Jellema et al., 2006). Disease etiology of SDAVF is unknown, but it is thought to be an acquired pathology that mainly affects middle-aged men (Jellema et al., 2006; Bretonnier et al., 2019).

The clinical manifestation of SDAVF is insidious and can often be overlooked during the initial examination (Koch, 2006; Steinmetz et al., 2004). The symptoms of SDAVF include a combination of gait disturbances, lower limb weakness, pain, sensory disturbances (paresthesia, hypesthesia, anesthesia, or hyperesthesia), as well as bowel and bladder dysfunction. The onset of these symptoms is progressive with a gradual deterioration over a period of 6 months to 2 years, although rapid deterioration has also been reported (Willinsky et al., 1990). The severity of clinical symptoms in SDAVF patients is influenced by multiple factors, with the American Spinal Injury Association Motor Score (ASIA-MS) showing a correlation to the extent of myelopathy prior to treatment (Filis et al., 2024a).

In the presence of clinical suspicion combined with radiological findings on magnetic resonance imaging (MRI), such as flow voids and myelopathy, digital subtraction angiography (DSA) is considered the gold standard for diagnosing and localizing the fistula point (Marcus et al., 2013). The treatment aim is to interrupt the fistulous arterial and venous point (Brinjikji et al., 2021; Oh et al., 2021; Acerbi and Ferroli, 2013; Kirsch et al., 2013; Krings and Geibprasert, 2009). Surgery represents the gold standard procedure, but endovascular therapy is an important alternative option (Cesak et al., 2018; Bretonnier et al., 2019; Takai et al., 2021; Filis et al., 2024b).

Both endovascular and surgical treatment have shown better neurological outcomes in several studies (Kirsch et al., 2013; Ropper et al., 2012), with surgical treatment being superior to embolization in terms of first occlusion and late recurrence rates in a meta-analysis of 1112 patients (96.6 % occlusion rate in the surgical group versus 72.2 % in the endovascular group) (Bakker et al., 2015). Both treatments result in improvements in symptoms such as pain, motor deficits, gait disturbances, and bowel and bladder dysfunction; however, they do not significantly relieve sensory disturbances (Filis et al., 2024b).

Several studies have shown that a longer duration of symptoms was associated with a worse neurological outcome (Tacconi et al., 1997; Ushikoshi et al., 1999; Shinoyama et al., 2010; Niimi et al., 1997; Safaee et al., 2018; Ronald et al., 2020; Ofran et al., 2013; Ruiz-et al., 2011), but some other studies have not been able to demonstrate such a correlation (Steinmetz et al., 2004; Safaee et al., 2018; Wojciechowski et al., 2017; Nagata et al., 2006; Lee et al., 2016; Durnford et al., 2017; Cenzato et al., 2004, 2012; Schuss et al., 2015; Kaufmann et al., 2011; Cecchi et al., 2008). In addition, a recent study found a significant correlation between the degree of reduction in spinal edema three months after surgery and the clinical outcome of patients after one year (Luo et al., 2022). Another study demonstrated that post-therapeutic MRI changes did not correlate with clinical outcomes and, therefore, cannot be regarded as a reliable prognostic indicator (Filis et al., 2024a). It remains unclear whether and which factors such as the duration of symptoms, the neurological functional status, the extent of myelopathy and flow voids on admission influence a patient's outcomes.

Due to the limited evidence and inconsistent results regarding prognostic factors in SDAVF patients, we investigated key preoperative variables that have not been comprehensively analyzed together in previous studies to identify which pretreatment characteristics might be associated with poorer outcomes following SDAVF treatment. In addition, we retrospectively analyzed the clinical characteristics and outcomes of SDAVF patients treated surgically and endovascularly at our institution over a 22-year period to identify the benefits and drawbacks of both treatments.

## 2 Methods

### 2.1 Study design

**Fig. 1:**
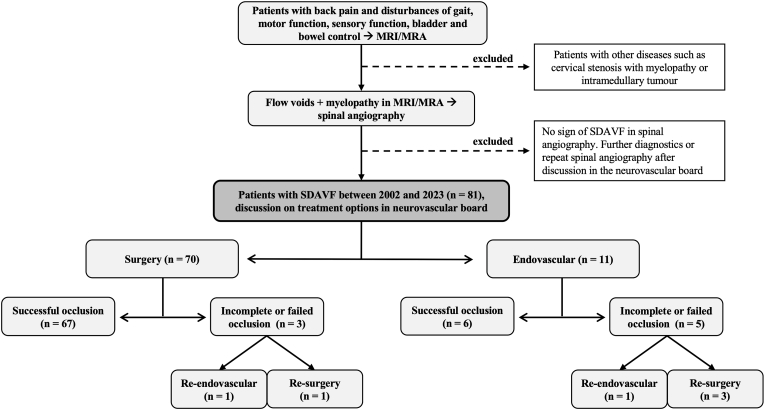
Study design. This figure shows our study design and flow charts for diagnosis of spinal dural arteriovenous fistula (SDAVF). MRI: magnetic resonance imaging; MRA: magnetic resonance angiography; DSA: digital subtraction angiography.

We performed a retrospective chart review of patients suffering from SDAVF and treated surgically or endovascularly at our neurosurgical-neuroradiological university center between 2002 and 2023. Eighty-one patients with SDAVF were identified, of whom seventy were treated surgically and eleven endovascularly. A total of 8 patients (3 surgical and 5 endovascular) had incomplete or failed SDAVF closure. Further surgical or endovascular treatment was performed in 6 patients, as 2 patients refused further treatment. Surgery was performed in 4 patients and endovascular treatment in 2 patients. Patients with suspected SDAVF on MRI (vascular myelopathy and flow voids), corresponding symptoms (gait dysfunction, sensory disturbances, motor deficits, bowel or bladder dysfunction, or back pain) and evidence of SDAVF on spinal DSA were included in the study. Patients without confirmed SDAVF were excluded. (see Fig. 1).

### 2.2 Patient data

The study was approved by the local ethics committee of the Carl Gustav Carus University Hospital in Dresden (Ref: BO-EK-437102023). Patients’ data were collected via the ORBIS system (ORBIS, Dedalus, Bonn, Germany) and neuroimaging studies through the IMPAX system (IMPAX, Impax Asset Management Group plc, London, UK). Radiological data, including MRI, magnetic resonance angiography (MRA), and DSA of the spine were available for review.

Electronic medical records were first pseudonymized and then analyzed for age, gender, time from first symptom to MRI-diagnosis, time from first MRI-diagnosis to first surgery or intervention, history of comorbidity (vascular disease, coronary heart disease, stroke, hypertension, degenerative spine disease, drugs that trigger bleeding, use of corticosteroids, and BMI), performance of a preoperative or postoperative MRI/MRA/DSA, number of incomplete or failed occlusions, number of secondary treatments performed (surgery or endovascular), treatment- or hospital-related complications, side of the fistula, location of the fistulous point, first symptom, neurological status pre-treatment, at the time of hospital discharge, first follow-up (3 months after discharge), second follow-up (6 months after discharge), third follow-up (12 months after discharge), ASIA-MS, and the modified Aminoff and Logue Scale (mALS).

Neurological status was assessed using the mALS for gait (range 0–5), urination (range 0–3), and defecation (range 0–3). Gait: grade 0 = normal; 1 = leg weakness, abnormal gait, or stance but no restriction of activity; 2 = restricted activity but not requiring support; 3 = requiring one stick for walking; 4 = requiring two sticks, crutches, or a walker; and 5 = confined to a wheelchair. Urination: grade 0 = normal; 1 = hesitancy, urgency, or frequency; 2 = occasional urinary incontinence or retention; and 3 = total incontinence or retention. Defecation: grade 0 = normal; 1 = mild constipation, responding well to aperients; 2 = occasional fecal dysfunction; and 3 = total fecal dysfunction. The mALS was determined as the sum of the gait, urination, and defecation grades, ranging from 0 to 11. A score of 0 indicates no functional deficits, while a score of 11 represents complete urinary and fecal incontinence as well as wheelchair dependency.

### 2.3 Clinical management

Diagnosis of SDAVF is based on clinical symptoms such as back pain, disturbances in gait, sensory, motor, bowel, or bladder function in association with myelopathy and flow voids on MRI/MRA. The diagnosis was confirmed by spinal DSA and each case was discussed in a multidisciplinary board with neurointerventional radiologists and neurosurgeons. If two therapeutic options were considered, the patient was usually informed and educated about both treatment options. The decision on the procedure was left to the patient. Endovascular treatment was preferred as a less invasive procedure in our hospital until around 2012. At that time, surgical treatment was suggested if endovascular treatment failed or was not feasible (vertebral artery or Adamkiewicz proximity with unintended risk of embolism). Since around 2012, surgical treatment has been the treatment of choice in our hospital and endovascular therapy has been considered as an alternative. Between 2002 and 2012, 10 of 42 patients underwent endovascular embolization. From 2013 onwards, no further embolizations were performed, except for one case in 2022 involving a fistula at S3 with bilateral feeders from the lateral sacral arteries, which was anatomically suited for embolization. In all cases, DSA and MRI/MRA were carefully reviewed by the neurosurgeon and a neurointerventional radiologist pretreatment to determine the exact location and side of the fistula. A postoperative spinal DSA was always performed after endovascular treatment and in most cases also MRA/MRI, whereby a DSA and/or MRI/MRA were conducted within the first 3 days after surgery to assess fistula closure and any postoperative complications. MRI/MRA was performed 3, 6 and 12 months after surgical or endovascular treatment to assess the disappearance of myelopathy and the regression of abnormal flow voids. The mean follow-up period was 22.4 months.

### 2.4 Statistical analysis

Statistical analysis of the data was performed using the SPSS software package (SPSS Statistics 29, IBM, Armonk, New York, USA). Descriptive statistics were used, and categorical variables were tested by Fisher exact tests or chi-square tests. Numerical variables were analyzed with Mann-Whitney U tests. All statistical tests were two-sided, and a p – value of less than 0.05 was considered statistically significant. The independent variable was the improved mALS over the disease course, and a backward multivariate binary logistic regression analysis was performed. In this way, the regression equation was established and the relative risk value for the mALS improvement in the clinical course (odds ratio) and the 95 % confidence interval (CI) were determined.

## 3 Results

### 3.1 Patient characteristics

Surgery was initially performed in 70 patients (86.4 %) and also in four patients in the case of incomplete or failed SDAVF closure. However, 11 patients (13.6 %) underwent initial endovascular treatment and 2 also received endovascular embolization after incomplete or failed closure of SDAVF. The surgical group (S) and the endovascular group (E) differed in age distribution (S: 64.9 ± 11.5 vs. E: 54.8 ± 16.4, p = 0.039) (Table 1). There was no gender-specific difference between the two groups. The time from symptom to MRI-diagnosis and from MRI-diagnosis to treatment did not differ between the two groups. The extent of myelopathy on MRI was similar in both groups (S: 7.1 ± 3.3 vs. E: 7.1 ± 2.3, p = 0.978), but the E group had more extensive flow voids compared to the S group (S: 6.9 ± 3.5 vs. E: 9.7 ± 3.1, p = 0.009).

**Table 1:** Baseline factors between surgical and endovascular treatment.

Variables	Surgical (n = 70, 86.4 %)	Endovascular (n = 11, 13.6 %)	p – value
Age, mean ± SD (years)	64.9 ± 11.5	54.8 ± 16.4	**0.039**^(2)^
Gender, n	F: 19, M: 51	F: 1, M: 10	0.227^(1)^
Time from symptom to diagnosis, mean ± SD (month)	10.9 ± 11.7	18.8 ± 17.3	0.152^(2)^
Time from diagnosis to treatment, mean ± SD (day)	21.7 ± 21.0	41.9 ± 39.3	0.142^(2)^
Myelopathy extension, mean ± SD (number of vertebrae)	7.1 ± 3.3	7.1 ± 2.3	0.978^(2)^
Flow voids extension, mean ± SD (number of vertebrae)	6.9 ± 3.5	9.7 ± 3.1	**0.009**^(2)^
MRI/MRA preoperative, n (%)	70/70 (100 %)	11/11 (100 %)	–
DSA preoperative, n (%)	70/70 (100 %)	11/11 (100 %)	–
MRI/MRA postoperative, n (%)	54/70 (77.1 %)	11/11 (100 %)	0.110^(1)^
DSA postoperative, n (%)	53/70 (75.7 %)	11/11 (100 %)	0.714^(1)^
Vascular disease, CHD, or stroke, n (%)	29/70 (41.4 %)	2/11 (18.2 %)	0.190^(1)^
Hypertension, n (%)	50/70 (71.4 %)	7/11 (63.6 %)	0.724^(1)^
Degenerative spine disease, n (%)	26/70 (37.1 %)	1/11 (9.1 %)	0.137^(1)^
Anticoagulants and antiplatelet agents, n (%)	19/70 (27.1 %)	2/11 (18.2 %)	0.677^(1)^
Use of corticosteroids, n (%)	13/70 (18.6 %)	2/11 (18.2 %)	1.0^(1)^
Obesity (BMI >30 kg/m^2^), n (%)	16/70 (22.9 %)	3/11 (27.3 %)	0.714^(1)^
First symptom:			
Pain, n (%)	12/70 (17.1 %)	3/11 (27.3 %)	0.495^(1)^
Motor deficits, n (%)	5/70 (7.1 %)	0/11 (0.0 %)	
Sensory disturbances, n (%)	19/70 (27.1 %)	5/11 (45.5 %)	
Gait disturbances, n (%)	33/70 (47.1 %)	3/11 (27.3 %)	
Bowel or bladder dysfunction, n (%)	1/70 (1.4 %)	0/11 (0.0 %)	
Fistulous point:			
Cervical, n (%)	3/70 (4.3 %)	1/11 (9.1 %)	**< 0.001**^(1)^
Upper thoracic, n (%)	10/70 (14.3 %)	2/11 (18.2 %)	
Lower thoracic, n (%)	35/70 (50.0 %)	1/11 (9.1 %)	
Lumbar, n (%)	20/70 (28.6 %)	3/11 (27.3 %)	
Sacral, n (%)	**2/70 (2.9 %)**	**4/11 (36.4 %)**	
Side of fistula, n	L:27, R:43, B:0	L:6, R:3, B:2	**< 0.001**^(1)^
Improvement in mALS, n (%)	45/70 (64.3 %)	3/11 (27.3 %)	**0.043**^(1)^
Incomplete or failed occlusion, n (%)	3/70 (4.3 %)	5/11 (45.5 %)	**< 0.001**^(1)^
Re-surgery or Re-intervention, n	RS: 1 vs. RI: 1	RS: 3 vs. RI: 1	**< 0.001**^(1)^
Treatment- or hospital-related complications, n (%)	15/70 (21.4 %)	1/11 (9.1 %)	0.542^(1)^

SD: Standard deviation; n: number; F: Female; M: Male; MRI: Magnetic resonance imaging; MRA: Magnetic resonance angiography; DSA: Digital subtraction angiography; CHD: Coronary heart disease; BMI: Body mass index; L: Left; R: Right; B: Both sides; mALS: modified Aminoff and Logue Scale; RS: Re-surgery; RI: Re-intervention; (1): Fisher exact test; (2): Mann–Whitney *U* test. Bold values are significant results (p < 0.05) as indicated in the methods.

Irrespective of their group affiliation, all patients received a preoperative MRA/MRI and a DSA, and there was also no difference between the two groups in terms of postoperative diagnostics (DSA, MRA/MRI). There was no difference between the two groups in terms of comorbidities such as hypertension, degenerative spinal diseases, use of anticoagulants and antiplatelet agents, use of corticosteroids, obesity (BMI >30 kg/m^2^), vascular disease, coronary heart disease or stroke. The distribution of the first symptoms was similar in both groups.

The spinal location of the fistulous point (cervical spine, upper thoracic spine, lower thoracic spine, lumbar spine, sacrum) was distributed differently in the two groups (p = 0.001). The side of the fistula was also different (S: left: 27, right: 43, both: 0 vs. E: left: 6, right: 3, both: 2, p < 0.001). A significantly higher proportion of surgical patients (64.3 %, 45/70) showed improvement in mALS compared to endovascular patients (27.3 %, 3/11; p = 0.043). The E group had a significantly higher rate of incomplete or failed closure than the S group (S: 3/70, 4.3 % vs. E: 5/11, 45.5 %, p < 0.001). Accordingly, the required re-operation or second endovascular treatment was greater in E group compared to S group (p < 0.001). There was no difference in terms of treatment- or hospital-related complications.

### 3.2 Symptoms and scores over disease course

The comparison between the two groups (S vs. E) with regard to the mALS and their single parameters such as gait (G), urination (U) and defecation (D) showed no significant differences in the clinical time course (preoperative, postoperative, at the first, second and third follow-up assessment) (Table 2). Furthermore, there was no difference between the two groups with regard to ASIA-MS, back pain, and sensory disturbances over time (Table 3).

**Table 2:** The modified Aminoff and Logue's Scale over the clinical course of disease.

Variables	Surgical (n = 70, 86.4 %)	Endovascular (n = 11, 13.6 %)	p - value^(1)^
mALS			
mALS preoperative, mean ± SD	5.0 ± 3.3	3.6 ± 2.9	0.183
mALS postoperative, mean ± SD	3.9 ± 3.3	4.2 ± 4.1	0.956
mALS at first follow up, mean ± SD	3.0 ± 2.8	4.4 ± 4.3	0.415
mALS at second follow up, mean ± SD	2.8 ± 2.6	4.9 ± 4.7	0.217
mALS at third follow up, mean ± SD	2.9 ± 2.8	5.4 ± 4.9	0.232

SD: Standard deviation; mALS modified Aminoff and Logue's Scale; (1): Mann–Whitney *U* test.

**Table 3:** ASIA-MS, pain, and Sensory disturbances over the clinical course of disease.

Variables	Surgical (n = 70, 86.4 %)	Endovascular (n = 11, 13.6 %)	p - value
ASIA-MS			
ASIA-MS preoperative, mean ± SD	89.0 ± 13.5	85.7 ± 19.2	0.748^(2)^
ASIA-MS postoperative, mean ± SD	93.3 ± 9.2	87.6 ± 19.2	0.470^(2)^
ASIA-MS at first follow up, mean ± SD	95.3 ± 6.8	88.4 ± 19.4	0.456^(2)^
ASIA-MS at second follow up, mean ± SD	95.9 ± 6.6	86.4 ± 20.2	0.215^(2)^
ASIA-MS at third follow up, mean ± SD	96.9 ± 6.4	84.9 ± 20.9	0.145^(2)^
Pain			
Pain preoperative, n (%)	40/70 (57.1 %)	7/11 (63.6 %)	0.754^(1)^
Pain postoperative, n (%)	18/70 (25.7 %)	2/11 (18.2 %)	0.723^(1)^
Pain at first follow up, n (%)	12/70 (19.0 %)	2/11 (18.2 %)	1.0^(1)^
Pain at second follow up, n (%)	12/59 (20.3 %)	3/10 (30.0 %)	0.679^(1)^
Pain at third follow up, n (%)	3/18 (16.7 %)	3/9 (33.3 %)	0.367^(1)^
Sensory disturbances			
Sensory disturbances preoperative, n (%)	56/70 (80.0 %)	10/11 (90.9 %)	0.360^(1)^
Sensory disturbances postoperative, n (%)	58/70 (82.9 %)	9/11 (81.8 %)	0.847^(1)^
Sensory disturbances at first follow up, n (%)	52/63 (82.5 %)	9/11 (81.8 %)	0.791^(1)^
Sensory disturbances at second follow up, n (%)	48/59 (81.4 %)	9/10 (90.0 %)	0.582^(1)^
Sensory disturbances at third follow up, n (%)	25/27 (94.4 %)	8/9 (88.98 %)	0.348^(1)^

ASIA-MS: American Spinal Injury Association motor score; SD: Standard deviation; n: number; (1): Fisher exact test; (2): Mann–Whitney *U* test.

### 3.3 Predictive factor

Based on the mALS values, we divided the patients into two subgroups (mALS improved vs. mALS not improved). The last documented mALS was compared with the preoperative mALS. If the last mALS is lower, the patient is classified as improved. Patients with an initial and final mALS of 0 were classified as improved, as no improvement is expected at 0. Patients with the same mALS on admission and at the last assessment as well as a mALS greater than one were categorized as not improved.

All factors that might have an independent influence on clinical outcomes, such as time interval between symptom and MRI diagnosis, time interval between MRI diagnosis and treatment, extent of myelopathy, extent of flow voids, preoperative ASIA-MS, preoperative mALS, obesity, and age, were first examined univariately and then analyzed in a backward multivariate binary logistic regression model.

The univariate analysis shows a significant value for Time from MRI diagnosis to treatment (0.978 (0.959–0.997); (OR (95 % CI)), p = 0.026) and for Time from symptom to MRI diagnosis (0.959 (0.924–0.996), p = 0.032), whereas in the multivariate binary logistic regression analysis only Time from diagnosis to treatment was significant (0.978 (0.959–0.997), p = 0.026) (Table 4).

**Table 4:** Univariate and multivariate logistic regression analysis to identify independent predictive factors.

Variables	Univariate logistic regression		Multivariate logistic regression	
	OR (95 % CI)	p value	OR (95 % CI)	p value
Age ≥70 years	1.310 (0.519–3.310)	0.567	1.435 (0.527–3.912)	0.480
Obesity (BMI >30 kg/m^2^)	0.702 (0.249–1.976)	0.503	0.801 (0.269–2.388)	0.691
Time from MRI diagnosis to treatment	0.978 (0.959–0.997)	**0.026**	0.978 (0.959–0.997)	**0.026**
Time from symptom to MRI diagnosis	0.959 (0.924–0.996)	**0.032**	0.969 (0.931–1.009)	0.130
Myelopathy extension (n of vertebrae)	1.005 (0.873–1.158)	0.941	0.999 (0.844–1.184)	0.993
Flow voids extension (n of vertebrae)	1.029 (0.906–1.169)	0.655	1.023 (0.888–1.177)	0.755
Preoperative ASIA-MS	1.005 (0.974–1.037)	0.747	1.004 (0.969–1.041)	0.805
Preoperative mALS ≥6	1.050 (0.419–2.632)	0.917	1.207 (0.399–3.649)	0.739

ASIA-MS: American Spinal Injury Association motor score; mALS modified Aminoff and Logue's Scale; BMI: Body mass index; MRI: Magnetic resonance imaging; n: number; OR: odds ratio, CI: confidence interval. Bold values are significant results (p < 0.05) as indicated in the methods.

## 4 Discussion

Our study provides insights into the clinical outcomes of surgical and endovascular treatment and predictive factor of rare SDAVF disease in a cohort of more than 80 patients over a period of more than 20 years in a specialized neurovascular and spine centre. We found that the time between MRI diagnosis and surgical or endovascular treatment significantly influenced clinical outcomes in terms of improvement in mALS between admission and the last follow up. Surgically treated patients showed a better initial occlusion rate of SDAVF and better long-term clinical outcomes in terms of mALS compared to endovascularly treated patients.

The higher success rate of surgical closure of SDAVF (95.7 %) compared to endovascular treatment (54.5 %) in our study was also reported in previous studies and meta-analysis (Cesak et al., 2018; Bretonnier et al., 2019; Bakker et al., 2015). With a low occlusion rate of endovascular therapy, it was to be expected that the second treatment in the E group would be higher than in the S group. In our studies, we found that the number of patients with an improvement in mALS in the clinical course (admission vs. last follow-up) was significantly higher with surgery than with endovascular treatment. This might be indirectly caused by the low initial occlusion rate of endovascular treatment, which was shown in the study by Bretonnier et al. (2019). On the contrary, Česák et al. was unable to demonstrate any clinical improvement in 24 surgically and interventional treated patients (Cesak et al., 2018).

The endovascularly treated patients in our cohort were about 10 years younger than the surgically treated and had extensive flow voids, although the extent of myelopathy was comparable between the two groups. The comorbidities, symptoms, complication rates, time from symptom onset to MRI diagnosis, and duration from MRI diagnosis to surgical or endovascular treatment were almost identical in the two groups. We do not believe that these factors influenced the results. The spinal location and side of the fistula differed between the two groups. Sacral and bilateral fistula were mainly treated endovascularly. We cannot exclude that these factors have no influence on the results of our studies.

The mALS and its single parameters such as gait, urination, and defecation as well as ASIA-MS, back pain, and sensory disturbances showed no differences between the two groups in the clinical time course (preoperative, postoperative, at the first, second and third follow-up examination). This supports that the improved mALS in the surgical group compared to endovascular group is only seen in the long-term course between admission and last follow up.

The univariate analysis showed that time delayed from MRI diagnosis to treatment (surgery or endovascular) and time delayed onset of symptoms to diagnosis on MRI were significantly different between the improved and non-improved mALS patient groups. We suggest that the time passed before treatment is very decisive for the clinical outcome and therefore divided the time between the time from onset of symptoms until MRI diagnosis and the time between MRI diagnosis and treatment. The time of MRI diagnosis was defined as the point at which SDAVF was suspected on MRI. Rapid worsening of insidious symptoms usually leads to an MRI with a suspected diagnosis of SDAVF. Consideration of all important preoperative factors such as age, obesity, time from diagnosis to treatment, time from symptom to diagnosis, extent of myelopathy, extent of flow voids, preoperative ASIA-MS, preoperative mALS in a multivariate logistic regression analysis model showed that time elapsed from MRI diagnosis to treatment was the most important independent factor for worse clinical outcome in relation to improvement in mALS.

### 4.1 Limitations and strengths of this study

The monocentric, retrospective nature of our analysis, the long inclusion interval, and the limited number of SDAVF patients treated with embolization (11 patients) might reduce the external validity of our study. Furthermore, our analysis could be affected by a possible selection bias due to our treatment flow charts, as our experience has been to favour surgery over embolization. Nevertheless, our cohort analysis is based on a 22-year treatment period of SDAVF in a large university neurosurgery centre, suggesting a high internal validity of our study. Therefore, our observations might be useful to understand the clinical course of SDAVF treated surgically or endovascularly.

## 5 Conclusions

Our study shows that surgical treatment of SDAVF is a safe and effective procedure compared with endovascular treatment, whereas endovascular treatment is an indispensable part of therapy in special cases such as sacral SDAVF regardless of the importance of spinal DSA in planning surgical treatment. The time between MRI diagnosis and surgical or endovascular treatment is crucial for the clinical outcome of SDAVF patients and should be shortened as possible to improve the clinical outcome of patients.

## Ethics approval and consent to participate

This study was reviewed and approved by the ethics committee of the University Hospital Dresden (Reference number: BO-EK-437102023). Written informed consent for participation in this study was not required according to national legislation and institutional requirements.

## Consent for publication

Patient consent was waived due to the retrospective, anonymous character of this study.

## Availability of data and materials

The datasets supporting the conclusions of this article are included within the article, further inquiries can be directed to the corresponding author.

## Funding

This research did not receive any specific grant from funding agencies in the public, commercial, or not-for-profit sectors.

## Declaration of competing interest

The authors declare that they have no known competing financial interests or personal relationships that could have appeared to influence the work reported in this paper.
