# Supplementary material for: Predictive factors and comparative analysis of surgical vs. endovascular treatment for spinal dural arteriovenous fistulas: a 22-year experience at a neurovascular and spine center
Source: Brain Spine. 2025 Jul 22;5:104335. doi: 10.1016/j.bas.2025.104335 (PMC12318290; doi:10.1016/j.bas.2025.104335)
Supplement: Multimedia component 1 [file mmc1.docx]

**Case illustration**

We present a 71-year-old female patient who complained to her general practitioner (GP) on 15 March about a feeling of weakness and tingling in her legs for about 14 days. The patient stated that these symptoms occur each time after walking more than 200 meters, so that she had to sit down. Her medical history included surgery and radio-chemotherapy for metastatic breast cancer 20 years ago and a subsequent diagnosis of polyneuropathy. There are no motor deficits in the upper and lower limbs and no back pain. Clinical signs of myelopathy were not investigated by the GP. Bladder and bowel dysfunction were denied. On the same day, an X-ray examination of the lumbar spine was performed to rule out bone metastases, the findings of which were unremarkable. In addition, an MRI of the lumbar spine to exclude spinal canal stenosis, a doppler sonography to exclude peripheral artery disease and an electrophysiological examination in the neurology department to ensure the absence of polyneuropathy were ordered. The MRI of the lumbar spine on the third of April showed a remarkable sign of myelopathy, so that the radiologist recommended an MRI of the cervical and thoracic spine. The GP arranged the diagnostics. On 20 April an MRI of the thoracic spine was performed with suspected SDAVF and on the 21st of April an MRI of the cervical spine was done without any abnormalities. However, the report was issued on 27 April. The case was presented to the neuroradiologist on 11 May and answered by the neuroradiologist on 15 May with the recommendation of a DSA. The patient agreed to be admitted to the neurosurgery department for a DSA and subsequent treatment on 23 May. The admission took place on 30 May with the DSA being performed. On admission, the patient showed gait disturbance, paresthesia after Th12 level, presence of the Babinski reflex on both sides, increased reflexes in the lower extremities, no bladder or bowel dysfunction, no radiculopathy, and no back pain. The DSA showed a SDAVF with a left-sided fistulous point at the level of Th10, myelopathy and flow voids from Th10 to 12 level. Subsequently, surgical treatment was performed without complications via hemilaminectomy of Th10 on the left on 2 June based on the decision of the neurovascular board. There was a postoperative improvement in the gait and sensory disturbances. On 7 June, the patient underwent a DSA, which showed a completely occluded SDAVF without evidence of any fistula and was discharged to home on 8 June. We wrote the date to reflect the delay from symptoms to diagnosis. In this case, it lasted about 3 months for the patient. On 12 December, the patient followed up with an MRI from November and showed a significant improvement in gait function but persistent paresthesia in both legs. The MRI on 14 November showed neither myelopathy nor flow voids. Pregabalin therapy was recommended and a further follow-up in 6 months (Figure 2).

**
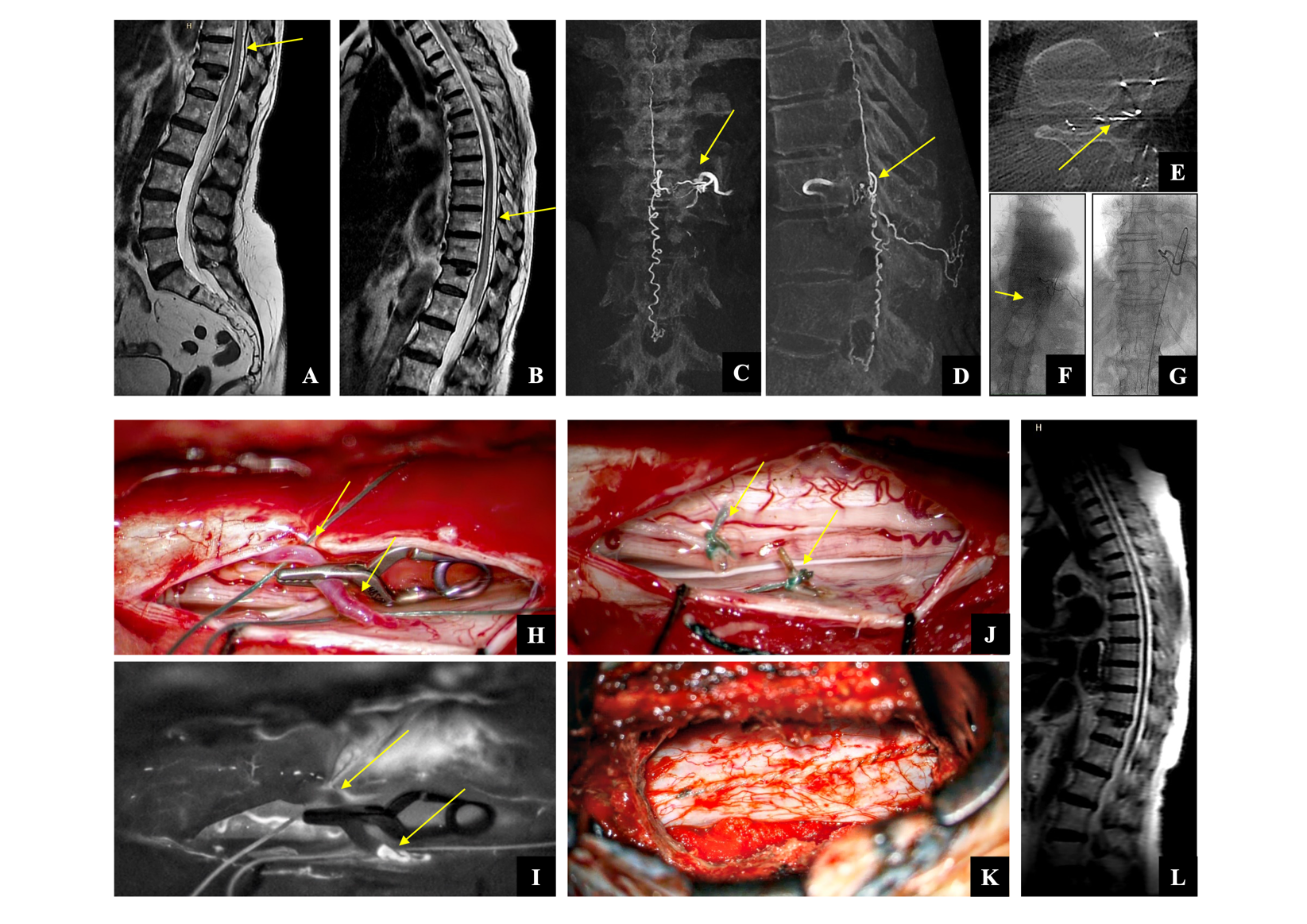
Figure 2: Case illustration of SDAVF treatment**

Case presentation of a left-sided spinal dural arteriovenous fistula (SDAVF) at Th 10 level. The sagittal T2-weighted magnetic resonance (MR) images (images A and B) show the myelopathy between the levels of Th 10 and Th 12 with flow voids (yellow arrows). The three-dimensionally reconstructed spinal digital subtraction angiography (DSA) (images C, D, and E) shows the fistulous point at Th 10 level on the left side as well as the flow voids (yellow arrows). Image F shows the SDAVF in the DSA preoperatively with flow voids (yellow arrow) and image G shows the absence of SDAVF postoperatively. Image H shows an intraoperative view after opening the dura and arachnoid with visualization of the fistula point and performing a temporary clipping (aneurysm clip), note the colors of the vessel before and after the clip (yellow arrows). Image I shows the use of indocyanine green fluorescein angiography (ICGFA). In the ICGFA the closure of the fistula is confirmed (yellow arrow). Image J shows the transected vessel after coagulation and ligation on both sides and removal of the clip. Image K showed the watertight dural suture. Image L presented the follow up sagittal T2-weighted MR-image, which showed the absence of myelopathy and flow voids.
